# Improvement in predicting drug sensitivity changes associated with protein mutations using a molecular dynamics based alchemical mutation method

**DOI:** 10.1038/s41598-020-58877-9

**Published:** 2020-02-07

**Authors:** Fumie Ono, Shuntaro Chiba, Yuta Isaka, Shigeyuki Matsumoto, Biao Ma, Ryohei Katayama, Mitsugu Araki, Yasushi Okuno

**Affiliations:** 1 Graduate School of Medicine, Kyoto University, 53 Shogoin-Kawaharacho, Sakyo-ku, Kyoto Japan; 2 Medical Sciences Innovation Hub Program, RIKEN, 1-7-22, Suehiro-cho, Tsurumi-ku, Yokohama, Kanagawa Japan; 3 Research and Development Group for In Silico Drug Discovery, Center for Cluster Development and Coordination (CCD), Foundation for Biomedical Research and Innovation at Kobe (FBRI) 6-3-5, Minatojima-Minamimachi Chuo-ku, Kobe Japan, Hyogo Japan; 4 Division of Experimental Chemotherapy, Cancer Chemotherapy Center, Japanese Foundation for Cancer Research, Tokyo, Japan

**Keywords:** Computational biophysics, Molecular medicine

## Abstract

While molecular-targeted drugs have demonstrated strong therapeutic efficacy against diverse diseases such as cancer and infection, the appearance of drug resistance associated with genetic variations in individual patients or pathogens has severely limited their clinical efficacy. Therefore, precision medicine approaches based on the personal genomic background provide promising strategies to enhance the effectiveness of molecular-targeted therapies. However, identifying drug resistance mutations in individuals by combining DNA sequencing and *in vitro* analyses is generally time consuming and costly. In contrast, *in silico* computation of protein-drug binding free energies allows for the rapid prediction of drug sensitivity changes associated with specific genetic mutations. Although conventional alchemical free energy computation methods have been used to quantify mutation-induced drug sensitivity changes in some protein targets, these methods are often adversely affected by free energy convergence. In this paper, we demonstrate significant improvements in prediction performance and free energy convergence by employing an alchemical mutation protocol, MutationFEP, which directly estimates binding free energy differences associated with protein mutations in three types of a protein and drug system. The superior performance of MutationFEP appears to be attributable to its more-moderate perturbation scheme. Therefore, this study provides a deeper level of insight into computer-assisted precision medicine.

## Introduction

Over the past three decades, molecular-targeted drugs have been developed for treating numerous diseases. However, the clinical efficacy of these drugs has been severely limited by mutations that impart drug resistance in target proteins. Mutation patterns can differ among individual patients with diseases such as cancer^1,2^, suggesting that precision medicine based on an individual’s genomic background offers a more-appropriate therapeutic strategy^3^. Analyses of personal genomes via DNA sequencing of tissue samples often reveal variants of unknown significance^4^ and multiple mutations^5^. Previously, the genotypes associated with drug responsiveness were identified from among mixtures of mutations through *in vitro* or *in vivo* functional studies, which are both time consuming and costly. Thus, an alternative method that enables rapid, precise, and accurate identification of drug-resistance mutations is required to facilitate the development of precision medicine therapies.

Recently, a database that comprises more than 1000 drug-resistance mutations has been developed^6^, and mutation-induced changes in drug affinity have been predicted by machine learning methods utilizing these big data^7,8^. Physicochemically, mutation-induced impairment of drug sensitivity can be defined as a difference in the protein-drug binding free energy (ΔΔ*G*) between $\Delta {{\rm{G}}}_{{\rm{b}}{\rm{i}}{\rm{n}}{\rm{d}}}^{1}$ for a wild-type target protein and $\Delta {{\rm{G}}}_{{\rm{b}}{\rm{i}}{\rm{n}}{\rm{d}}}^{2}$ for a specific mutant. Molecular dynamics (MD)-based free energy computation methods such as free energy perturbation (FEP)^9–11^, have been employed in genomic medicine studies^12–17^. One alchemical FEP method in particular, MP-CAFEE (massively parallel computation of absolute binding free energy with well-equilibrated states)^18^, was used to predict decreases in drug sensitivity resulting from mutations in anaplastic lymphoma receptor tyrosine kinase (ALK)^13,14^, *RET* proto-oncogene products (RET)^15^, and the epidermal growth factor receptor (EGFR)^16^. However, Δ*G* values computed using MP-CAFEE often exhibit larger calculation errors resulting from the method’s FEP scheme, a double-annihilation method^19^. In this scheme, intermolecular interactions between a drug and its surrounding molecules are gradually annihilated, such that in the final stages of annihilation (when the coupling parameter, λ, is ~1), the drug leaves the protein pocket and freely moves within the simulation box, leading to difficulty in determining free energy convergence during short MD simulations (i.e., the end-point problem)^20,21^. Although several studies have implemented improvements in convergence by introducing artificial restraints that confine the drug within the binding pocket^19,22^, use of these methods may require additional effort in order to determine the proper restraint parameters.

The present study compared MP-CAFEE and an alternative FEP protocol based on an alchemical mutation algorithm^23^, MutationFEP, in terms of performance in predicting mutation-induced changes in drug sensitivity using three protein systems: ALK with ALK tyrosine kinase inhibitor (alectinib), a viral protein, H1N1–2009 neuraminidase (NA) with neuraminidase inhibitor (oseltamivir), and aldose reductase (ALR2) with five drugs. Because the latter protocol only perturbs intermolecular interactions involving the mutated residue(s), most protein-drug interactions are maintained during FEP simulations, thus potentially avoiding the end-point problem. The use of MutationFEP significantly improved the free energy convergence with better prediction performance, demonstrating that MutationFEP is not subject to the intrinsic drawbacks associated with conventional FEP methods. MutationFEP is thus expected to become an invaluable computational tool that could accelerate the development of new precision medicine therapies.

## Materials and Methods

### Preparation of initial structures

We calculated binding free energy differences between the wild-type and mutant forms of three proteins, ALK (mutations: I1171T, I1171N, F1174I, F1174V, V1180L, V1185L, L1196M, L1196Q, and G1269A), NA (mutations: I223V, S247N, H275Y, I223V/H275Y, and S247N/H275Y) and ALR2 (mutations: V47I, T113Y, L300A, L301M, and S302R/C303D). The ALK-alectinib cocrystal structure was obtained from the Protein Data Bank (PDB)^24^ (code 3AOX^25^). The NA-oseltamivir cocrystal structure was obtained from the PDB (code 3TI6^26^), and chain A of the tetramer in the deposited structure was used in the subsequent structure preparation. Crystal structures of ALR2 in complex with zopolrestat, fidarestat, IDD388, 47D, and IDD393 were obtained from the PDB (codes 2HVO^27^, 1PWM^28^, 2IKI^29^, 2PDG^30^, and 2PZN **(**Ruiz, F. *et al*. to be published**)**, respectively).

After small-molecule compounds other than drug molecules or a cofactor (NADP^+^) were removed from the PDB structures, the disordered loops and flexible side chains in the proteins were modeled using the Structure Preparation Module of Molecular Operating Environment (MOE, Chemical Computing Group, Montreal, Canada), version 2013.08. The N- and C-termini of the protein model were capped with acetyl and N-methyl groups, respectively. For MP-CAFEE, each mutation was introduced into the structure of wild-type NA, ALK, and ALR2 using the Structure Preparation Module in MOE. Hydrogen atoms of a protein were added using pdb2gmx, a module of the GROMACS program, v. 5.1.4^31^. The AMBER ff99SB-ILDN^32^ force field was used for protein and ion molecules. Hydrogen atoms of drugs were added as described in Supplementary Fig. S1. General Amber force field (GAFF)^33^ was used for drug molecules. The restrained electrostatic potential (RESP) approach^34^ was applied to determine partial atomic charges of a drug using the resp module of AMBERTools 13 after its structure was optimized in vacuo and the electrostatic potential was calculated at the HF/6–31G(d) level using the General Atomic and Molecular Electronic Structure System (GAMESS version 14 Feb 2018 [R1])^35^. Topology and coordinate files for the drug were generated by using tleap module of AMBERTools 13 and converted to GROMACS-compatible files with acpype^36^.

The protein-drug complex was placed 0.8 nm from the end of the periodic octahedron box filled with water molecules, for which TIP3P^37^ was used. Several water molecules were replaced by sodium and chloride ions to neutralize the system using the genion module of GROMACS. The simulation systems constructed according to these procedures contained approximately 32,700, 41,700, and 30,000 atoms for NA, ALK, and ALR2, respectively, and were used as the initial structures in subsequent MD simulations. The determined parameters and input coordinates are available as Supplemental Data.

### MD simulation

First, in order to eliminate steric clashes, energy minimizations using the steepest descent algorithm were performed. Each of the systems was then equilibrated at five different initial velocities in accordance with algorithms and settings described below unless otherwise noted. The detailed procedure was as follows: (1) a 100-ps constant volume (NVT) ensemble simulation with the restraints; (2) a 100-ps constant pressure (NPT) ensemble simulation with the restraints; and (3) a 50-ns NPT ensemble simulation without the restraints were sequentially performed.

The initial structure for following free energy calculations was selected from among snapshots obtained from the 50-ns × 5 simulations, according to the procedure employed in a previous study^38^.

All minimization and MD simulations were performed using GROMACS 5.1.4. Coulomb interactions were calculated according to the particle mesh Ewald^39^ method; the real-space cut-off value was set to 1.0 nm, with 72, 72, and 72, and 64, 64, and 64 wave vectors included for the x, y, and z directions for the reciprocal space calculations for the ALK and ALR2 systems, and NA system, respectively. The β–spline interpolation order was set at 4. The cut-off value of vdW interactions was set to 1.0 nm. The vdW and Coulomb interaction energies were shifted to 0 at the cut-off radius. SETTLE^40^ was used for the rigid water model. P-LINCS^41^ was applied to constrain all bond lengths at an expansion order of 6 for production runs and 8 for other runs. The V-rescale^42^ and Parrinello-Rahman algorithms^43^ were used to control the temperature at 300 K and pressure at 1.01325 bar, respectively. Position restraints were controlled by the harmonic potential at a force constant of 1000 kJ/(mol nm^2^).

### Calculation of protein-drug binding free energies

**Figure 1:**
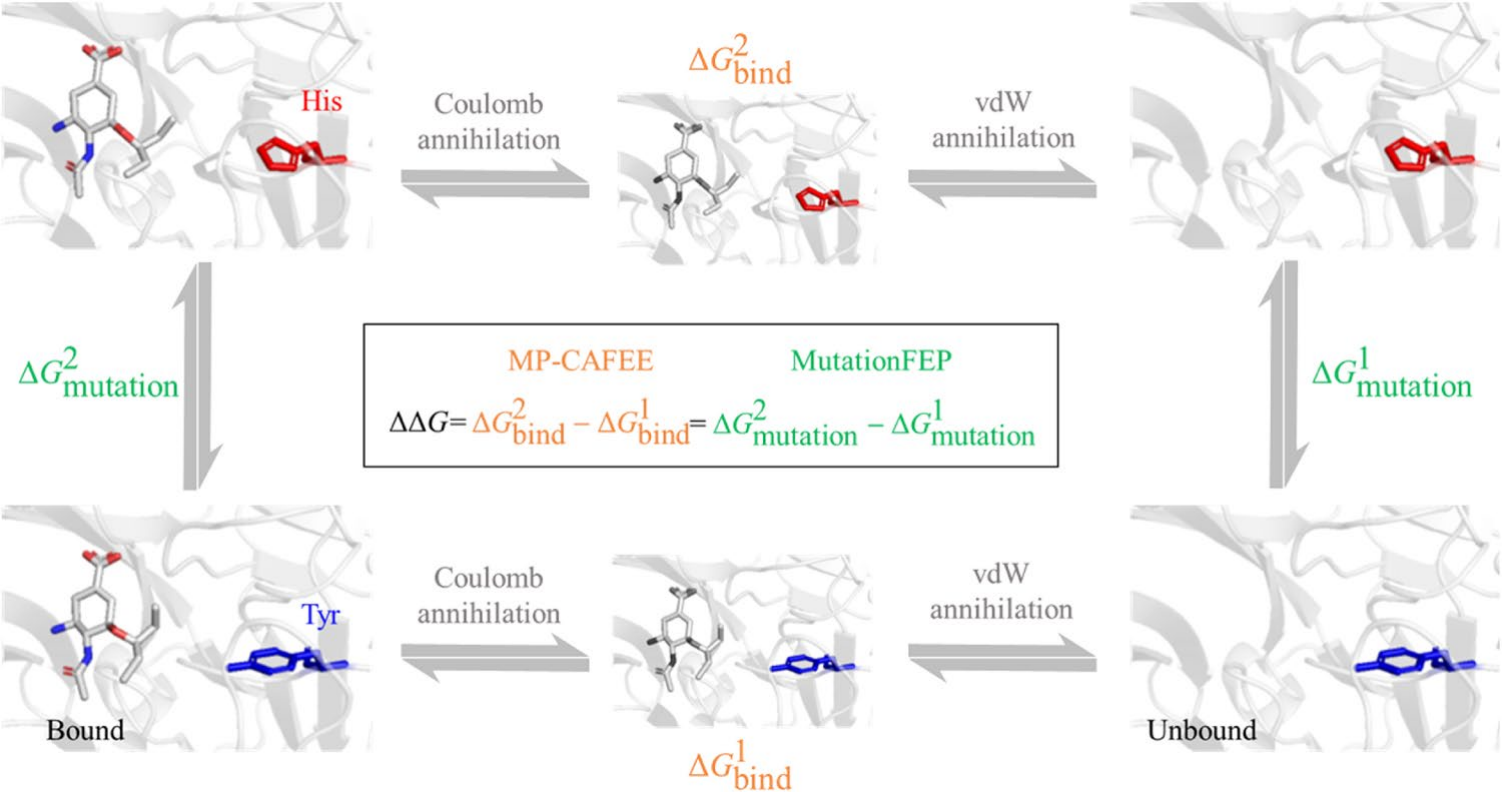
Difference in free energy calculated using MP-CAFEE or MutationFEP. In MP-CAFEE $(\Delta \Delta {{\rm{G}}}_{\text{MP}-\text{CAFEE}}\,=\,\Delta {{\rm{G}}}_{{\rm{b}}{\rm{i}}{\rm{n}}{\rm{d}}}^{2}\,-\,\Delta {{\rm{G}}}_{{\rm{b}}{\rm{i}}{\rm{n}}{\rm{d}}}^{1})$, ΔΔG is computed as the difference between $\Delta {{\rm{G}}}_{{\rm{b}}{\rm{i}}{\rm{n}}{\rm{d}}}^{1}$ for the wild-type state and $\Delta {{\rm{G}}}_{{\rm{b}}{\rm{i}}{\rm{n}}{\rm{d}}}^{2}$ for the mutant, where Coulomb and van der Waals (vdW) interactions between a drug and other molecules are gradually annihilated. In MutationFEP $(\Delta \Delta {{\rm{G}}}_{{\rm{M}}{\rm{u}}{\rm{t}}{\rm{a}}{\rm{t}}{\rm{i}}{\rm{o}}{\rm{n}}{\rm{F}}{\rm{E}}{\rm{P}}}\,=\,\Delta {{\rm{G}}}_{{\rm{m}}{\rm{u}}{\rm{t}}{\rm{a}}{\rm{t}}{\rm{i}}{\rm{o}}{\rm{n}}}^{2}\,-\,\Delta {{\rm{G}}}_{{\rm{m}}{\rm{u}}{\rm{t}}{\rm{a}}{\rm{t}}{\rm{i}}{\rm{o}}{\rm{n}}}^{1})$, ΔΔG is computed as the difference between two different mutation-induced free energy changes: $\Delta {{\rm{G}}}_{{\rm{m}}{\rm{u}}{\rm{t}}{\rm{a}}{\rm{t}}{\rm{i}}{\rm{o}}{\rm{n}}}^{1}$ for the drug-free state, and $\Delta {{\rm{G}}}_{{\rm{m}}{\rm{u}}{\rm{t}}{\rm{a}}{\rm{t}}{\rm{i}}{\rm{o}}{\rm{n}}}^{2}$ for the drug-bound state.

Binding free energy differences between wild-type and mutant proteins were calculated using two alchemical FEP methods, as shown in Fig. 1 (i.e., MutationFEP and MP-CAFEE).

MutationFEP. MutationFEP calculations were performed according to the dual-topology method^44^, in which systems were altered using a coupling parameter, *λ*, ranging from *λ* = 0 (corresponding to a wild-type protein) to *λ* = 1 (corresponding to a mutant protein). Protein dual topologies were prepared using pmx (1.1.0dev)^23,45^, a structure and topology generator for FEP. For topologies of NA, the atomic weight of dummy atoms was set to 1 to prevent topological instability in the FEP simulations. The equilibrated systems prepared in the previous section were used for simulations of the drug-bound state. Simulations of the drug-free state were performed using a protein structure generated by removing the drug from the drug-bound state (Fig. 1). In our protocol, 8, 14, and 27 *λ* points were used, as shown in Supplementary Table S1. After each *λ* system was energy minimized using the steepest-descent and l-BFGS algorithms, it was equilibrated by performing (1) a 10-ps NVT simulation, (2) a 10-ps NPT simulation (using the Berendsen barostat), and (3) a 30-ps NPT simulation, with positional restraints of protein heavy atoms, except for mutated residues. The V-rescale^42^ and Parrinello-Rahman algorithms^43^ were used to control the temperature at 300 K and pressure at 1.01325 bar, respectively, for all systems, except for the first 30-ps NPT ensemble simulation of MutationFEP, which was controlled using the Berendsen barostat^46^. Three independent production runs were conducted at different initial velocities under the NPT condition without the positional restraints. After the first 2 ns in each trajectory were discarded, the binding free energies were calculated by the multistate Bennett acceptance ratio (MBAR) method^47^, using the alchemical-analysis.py module (1.0.2.dev0)^48^. Calculation errors in $\Delta \Delta {{\rm{G}}}_{{\rm{M}}{\rm{u}}{\rm{t}}{\rm{a}}{\rm{t}}{\rm{i}}{\rm{o}}{\rm{n}}{\rm{F}}{\rm{E}}{\rm{P}}}$ were estimated according to the following equation:

$${\sigma }_{{\rm{m}}{\rm{u}}{\rm{t}}{\rm{a}}{\rm{t}}{\rm{i}}{\rm{o}}{\rm{n}}}^{2-1}\,=\,\sqrt{{({\sigma }_{{\rm{m}}{\rm{u}}{\rm{t}}{\rm{a}}{\rm{t}}{\rm{i}}{\rm{o}}{\rm{n}}}^{2})}^{2}\,+\,{({\sigma }_{{\rm{m}}{\rm{u}}{\rm{t}}{\rm{a}}{\rm{t}}{\rm{i}}{\rm{o}}{\rm{n}}}^{1})}^{2}},$$

where ${\sigma }_{{\rm{mutation}}}^{1}\,{\rm{and}}\,{\sigma }_{{\rm{mutation}}}^{2}$represent the standard deviation of $\Delta {{\rm{G}}}_{{\rm{m}}{\rm{u}}{\rm{t}}{\rm{a}}{\rm{t}}{\rm{i}}{\rm{o}}{\rm{n}}}^{1}$ and $\Delta {{\rm{G}}}_{{\rm{m}}{\rm{u}}{\rm{t}}{\rm{a}}{\rm{t}}{\rm{i}}{\rm{o}}{\rm{n}}}^{2}$ respectively, across three independent simulations.

### MP-CAFEE

MP-CAFEE was performed with the previously determined simulation parameters^38^, in which 11 and 21 *λ* points were used to decouple Coulomb and van der Waals interactions between drug and other molecules, respectively. For each *λ*, six independent 2-ns simulations were performed at different initial velocities. After the first 1 ns in each trajectory was discarded, the binding free energies were calculated by the MBAR method. Calculation errors in ΔΔ*G*_MP-CAFEE_ were estimated as the standard deviation of $\Delta {{\rm{G}}}_{{\rm{b}}{\rm{i}}{\rm{n}}{\rm{d}}}^{2}$ across the six independent simulations (Fig. 1).

## Results

### Parameter optimization in MutationFEP

**Figure 2:**
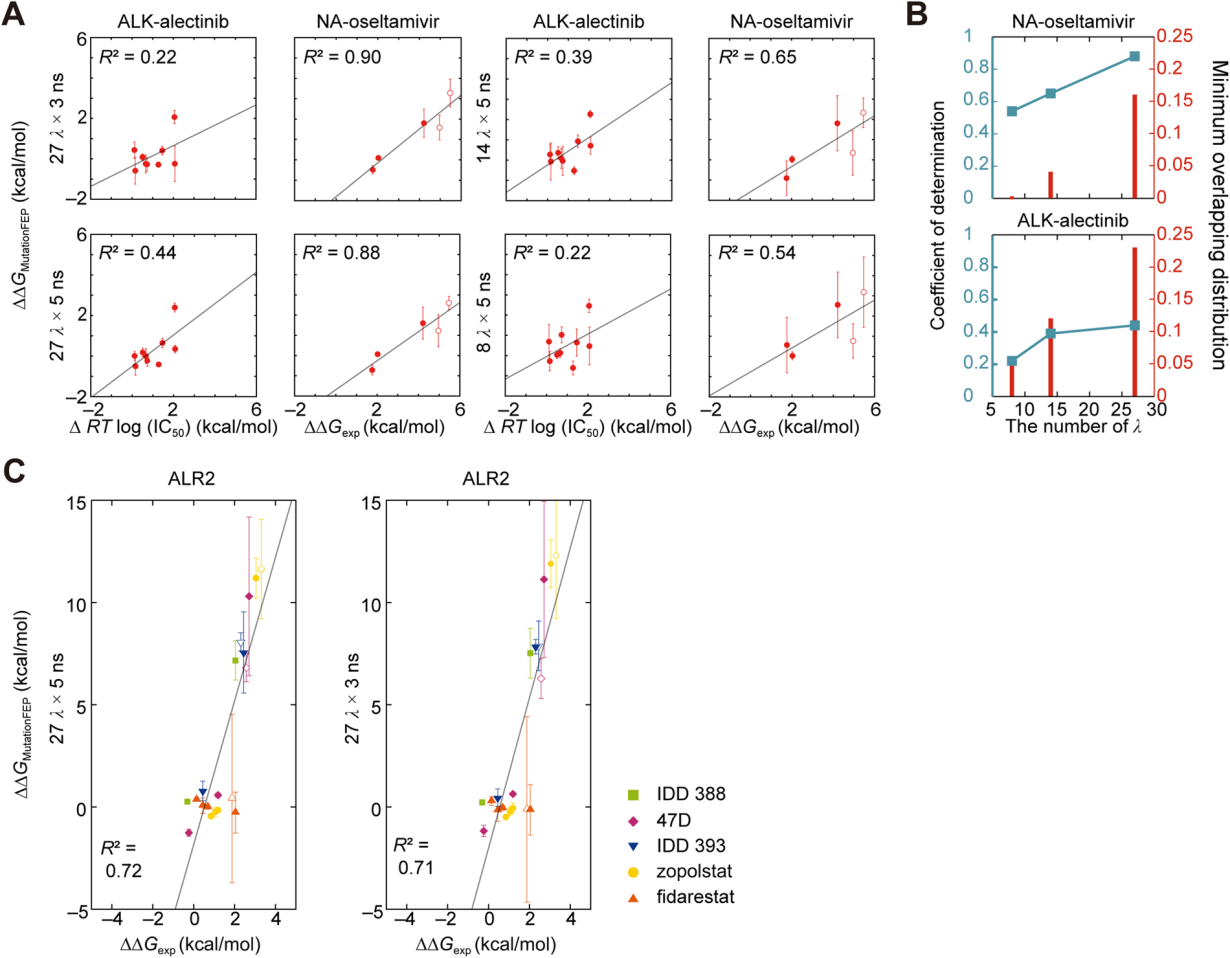
Performance of MutationFEP. (**A**) Calculated mutation-induced free energy changes $(\Delta \Delta {{\rm{G}}}_{{\rm{M}}{\rm{u}}{\rm{t}}{\rm{a}}{\rm{t}}{\rm{i}}{\rm{o}}{\rm{n}}{\rm{F}}{\rm{E}}{\rm{P}}}\,=\,\Delta {{\rm{G}}}_{{\rm{m}}{\rm{u}}{\rm{t}}{\rm{a}}{\rm{t}}{\rm{i}}{\rm{o}}{\rm{n}}}^{2}\,-\,\Delta {{\rm{G}}}_{{\rm{m}}{\rm{u}}{\rm{t}}{\rm{a}}{\rm{t}}{\rm{i}}{\rm{o}}{\rm{n}}}^{1})$ versus experimentally determined drug sensitivity changes. The coefficient of determination, *R*^2^, was calculated by linear regression (gray lines). Open symbols indicate double point mutations. These calculated and experimental values are summarized in Supplementary Tables S2A-B. (**B**) The dependence of *R*^2^ (blue lines) and the minimum energy overlap (red bars) on the number of *λ*. Five-nanosecond trajectories were used for calculation of these values. Experimental values were retrieved from *K*_i_ values of oseltamivir for NA in the enzymatic assays^53^ and IC_50_ values of alectinib for ALK mutations in the cell viability assays^14^. Those for ALK wild-type, G1269A, and F1174I mutations were determined by the identical procedure in this study. (**C**) The performance for the aldose reductase (ALR2) systems. Open symbols indicate double point mutations. Experimental values of five drugs (IDD388, 47D, IDD393, zopolrestat, fidarestat) were retrieved from Δ*G* values^30^. These calculated and experimental values are summarized in Supplementary Table S2C. Error bars represent the standard deviation across three independent FEP simulations.

We examined the effect of MD simulation time (*t* ns) and number of *λ* (*n λ*) on prediction performance and calculation errors. The coefficients of determination (*R*^2^) for the ALK-alectinib and NA-oseltamivir systems were 0.22 and 0.90, respectively, for (27 *λ* × 3 ns) and 0.44 and 0.88, respectively, for (27 *λ* × 5 ns) (Fig. 2A). In the ALK-alectinib system, longer simulation time resulted in increased *R*^2^ values with lower calculation errors, similar to several proteins assessed in previous studies^49^. On the other hand, there was no significant difference for the NA-oseltamivir system. These results suggest that the appropriate length for the simulation time differs depending on protein species. Furthermore, when the simulation length was fixed at 5 ns, the prediction performance improved as the number of *λ* increased (Fig. 2B). A previous study suggested that an energy overlap between neighboring *λ*-states of more than 0.03 is preferable to avoid increasing the calculation error^48^. Our simulations at 14 *λ* and 27 *λ* maintained an energy overlap of more than 0.03, but the overlap in simulations at 8 *λ* decreased below 0.03 and resulted in a marked increase in the calculation error (Fig. 2A). A typical example of energy overlap is shown in Fig. S2.

### Performance of MutationFEP

A protocol with the best performance (27 × 5 ns) was applied to the aldose reductase-drug system consisting of 2–5 mutants and 5 drugs, showing a coefficient of determination of 0.72 (Fig. 2C). It took 48 hours to obtain 19 ΔΔ*G* values when using 128 nodes, each of which is equipped with 2 CPUs (Xeon Gold 6148 CPU, 20 cores). Parallel computing enabled simultaneous prediction of ΔΔ*G* for multiple mutants.

### Comparison of the performance of MP-CAFEE and MutationFEP

**Figure 3:**
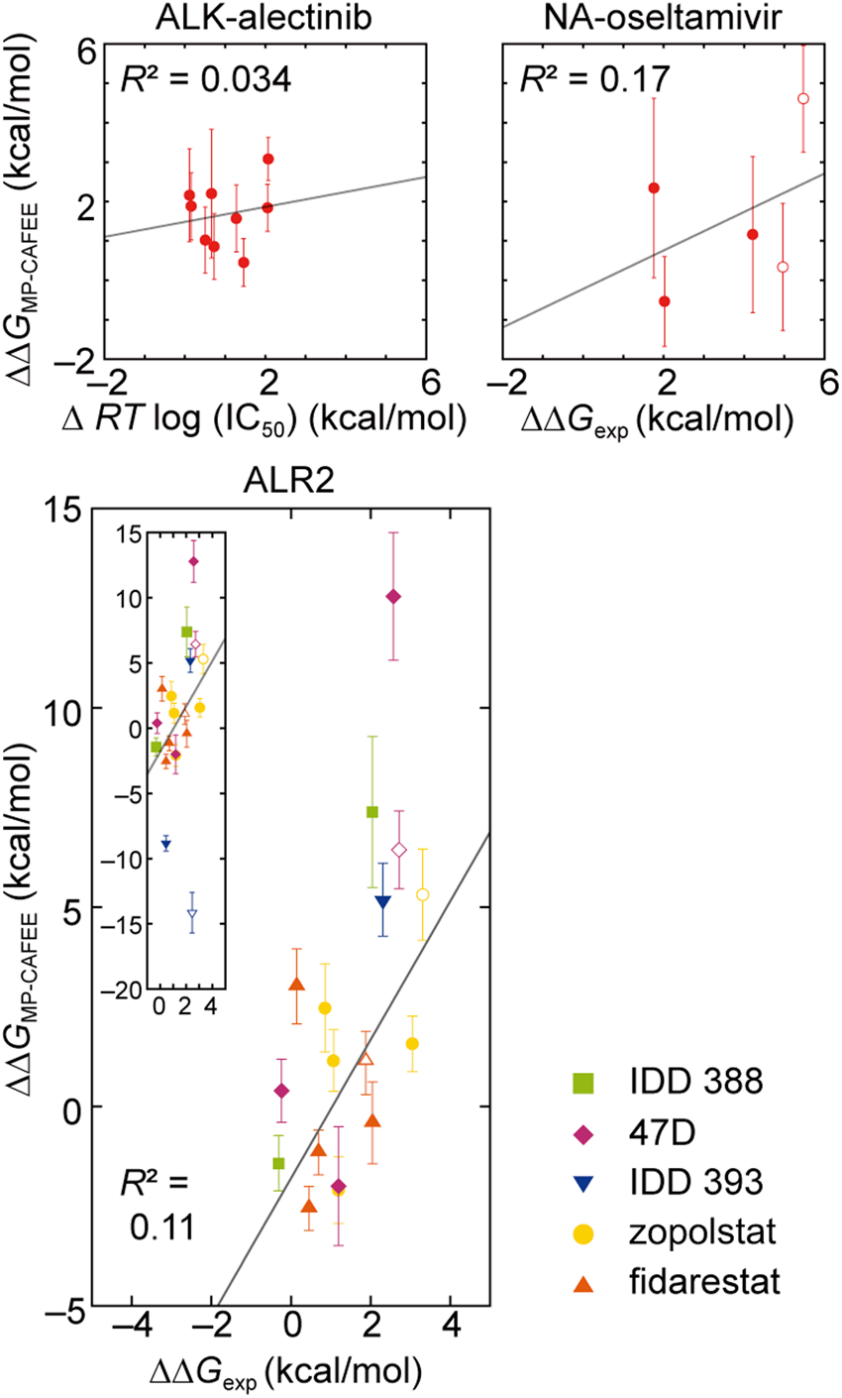
Performance of MP-CAFEE. Calculated mutation-induced free energy changes $(\,\Delta \Delta {{\rm{G}}}_{{\rm{M}}{\rm{P}}-{\rm{C}}{\rm{A}}{\rm{F}}{\rm{E}}{\rm{E}}}\,=\,\Delta {{\rm{G}}}_{{\rm{b}}{\rm{i}}{\rm{n}}{\rm{d}}}^{2}\,-\,\Delta {{\rm{G}}}_{{\rm{b}}{\rm{i}}{\rm{n}}{\rm{d}}}^{1})$ are plotted against experimentally determined drug sensitivity changes. The coefficient of determination, *R*^2^, was calculated by linear regression (gray lines). Open symbols indicate double point mutations. Experimental values were retrieved as described in Fig. 2. Error bars represent the standard deviation across six independent FEP simulations. For the ALK L1196M mutant, since the MBAR algorithm (an alchemical analysis calculation module) did not converge, only five of the six simulations were used for Δ*G* calculation. These calculated and experimental values are summarized in Supplementary Tables S2A–C.

When we performed MP-CAFEE to calculate ΔΔ*G* for the same protein-drug systems, the *R*^2^ values for the ALK-alectinib, NA-oseltamivir, and ALR2–5 drug systems were 0.034, 0.17, and 0.11, respectively (Fig. 3). These *R*^2^ values were significantly smaller than those of MutationFEP (27 *λ* × 3 ns) (Fig. 2A,C). The computational cost was almost equivalent to that of MP-CAFEE, thus demonstrating the higher prediction performance of MutationFEP (Supplementary Tables S2C). Moreover, MP-CAFEE exhibited larger calculation errors compared to MutationFEP (Figs. 2A,C, and 3).

## Discussion

**Figure 4:**
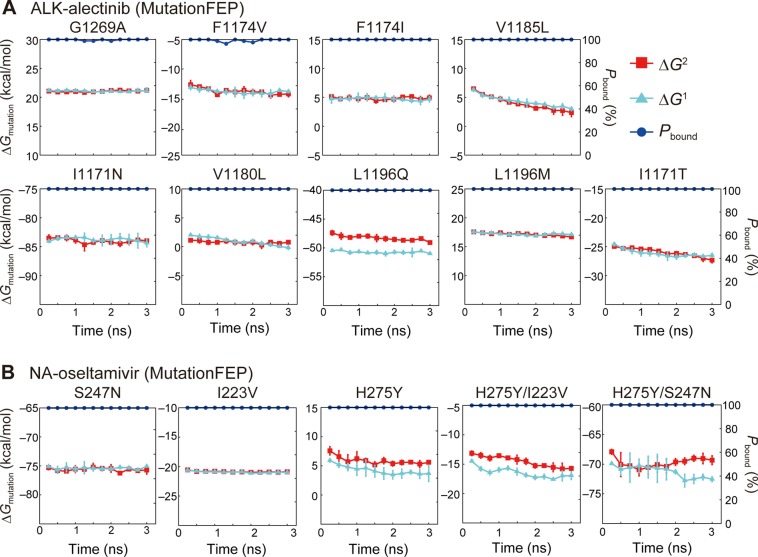
Free energy convergences in MutationFEP for the (**A**) ALK-alectinib and (**B**) NA-oseltamivir systems. Free energy values were computed using (27 *λ* × 3 ns) trajectories. Error bars indicate the standard deviation across three independent simulations. The proportion of trajectories that maintained the drug-bound state, *P*_bound_ (%), was calculated as follows. The average distance between the centers of mass of the protein and drug (COM distance) was measured for each *λ* trajectory, and when the COM distance was less than a given threshold, the trajectory was regarded as maintaining the bound state. *P*_bound_ was calculated by dividing the number of *λ* trajectories that maintained the bound state by that of all *λ* trajectories. The threshold for each protein, *T*, was determined according to the equation *T* = *ave*. + 6 × *s.t.d*, where *ave*. and *s.t.d*. are the average and standard deviation of the COM distances across the three independent unperturbed (*λ* = 0) simulations.

**Figure 5:**
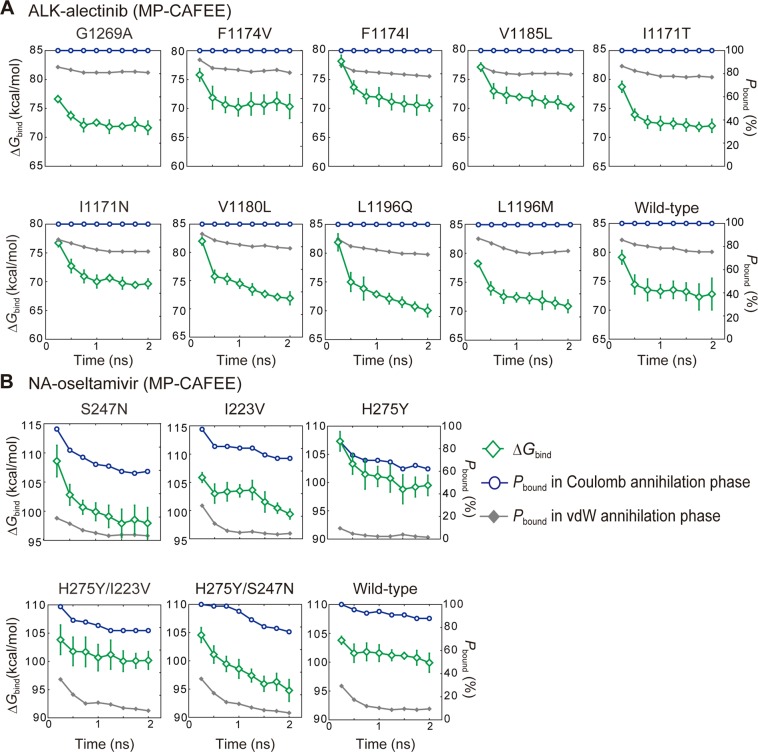
Free energy convergences in MP-CAFEE for the (**A**) ALK-alectinib and (**B**) NA-oseltamivir systems. Free energy values were computed using trajectories of the Coulomb and vdW annihilation phases. Error bars indicate the standard deviation across six independent simulations. The proportion of trajectories that maintained the drug-bound state, *P*_bound_ (%), was calculated as described in Fig. 4. A COM distance threshold was determined using the same equation in Fig. 4, where *ave*. and *s.t.d*. are set to the average and standard deviation of the COM distances across the six independent unperturbed (*λ* = 0) simulations.

**Figure 6:**
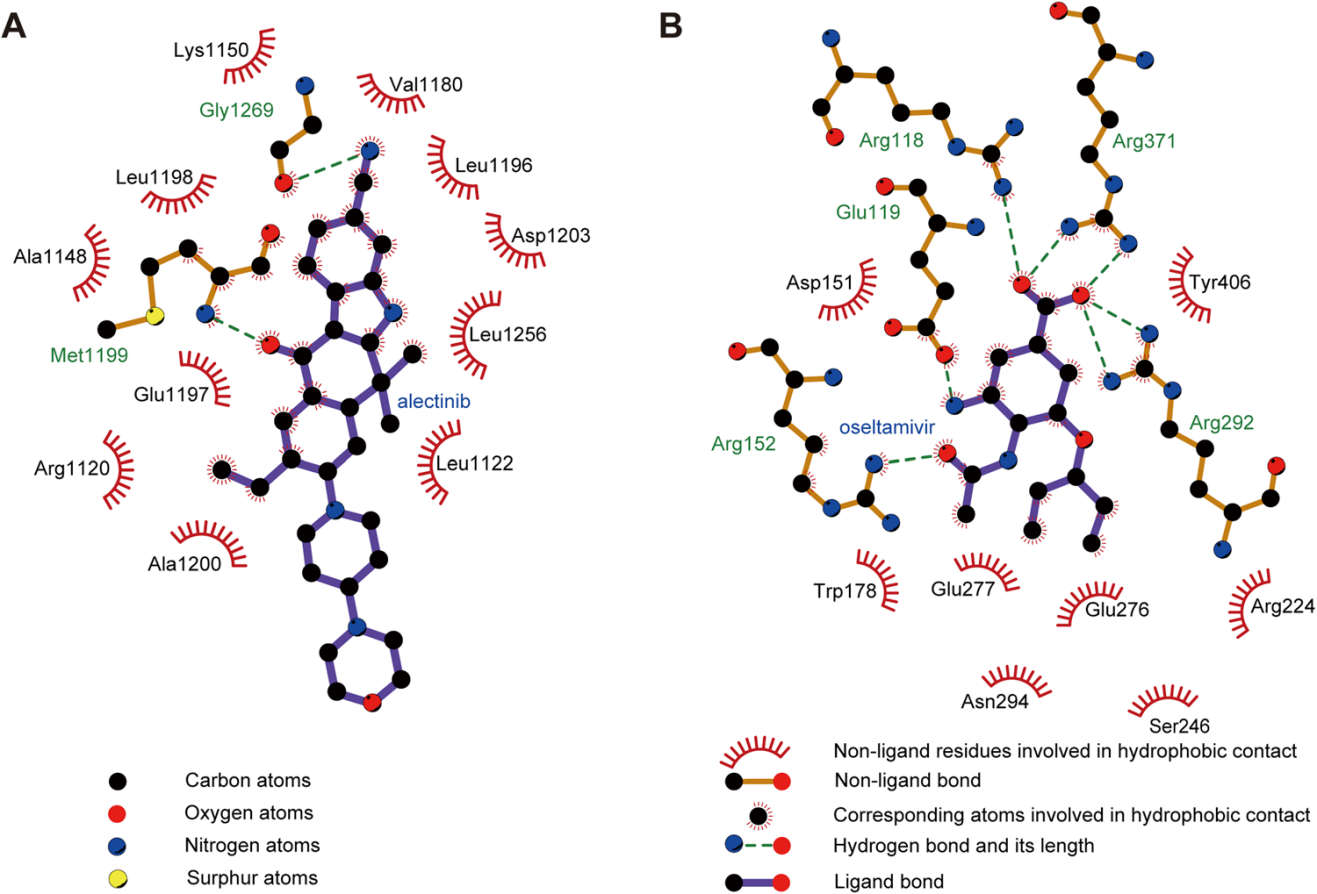
Interactions between drug and protein residues in the binding pocket. The cocrystal structures of (**A**) ALK-alectinib (PDB ID: 3AOX)^25^ and (**B**) NA-oseltamivir (PDB ID: 3TI6)^26^ were analyzed using LigPlot + ^54^. The red half circles denote the residues that formed hydrophobic interactions, and the green dotted lines represent the hydrogen bonds.

In this study, we employed an alchemical mutation FEP protocol, MutationFEP, to predict mutation-induced changes in drug sensitivity for three different proteins, NA, ALK, and ALR2. We found that MutationFEP provides better prediction performance in terms of ΔΔ*G* (Figs. 2 and 3) than a conventional FEP method, MP-CAFEE. We next examined factors that cause the performance difference between the two methods, using the simulation data of the NA and ALK systems. MutationFEP showed lower calculation errors (Figs. 2 and 3) and better Δ*G* convergences (Figs. 4 and 5) than MP-CAFEE. In the MP-CAFEE scheme, the bound drug tends not to stay in the binding pocket at the end point of the perturbation (*λ* ~ 1), leading to not only enhanced motional freedom of the drug, but also conformational changes in the protein upon drug dissociation^19,21^. This situation may lead to sampling insufficiency in short MD simulations. Indeed, drug dissociation in the vdW annihilation phase was clearly observed for both proteins (Fig. 5). Unexpectedly, in the NA-oseltamivir system, drug dissociation was observed even in the Coulomb annihilation phase, corresponding to the early stage of the perturbation (Fig. 5B), presumably because bound oseltamivir is heavily stabilized by electrostatic interactions (i.e., hydrogen bonds and salt bridges) (Fig. 6). In contrast, MutationFEP indicated stable maintenance of the drug-bound state for both proteins (Fig. 4), as its FEP scheme perturbs only the mutated amino acid(s), thus avoiding the end-point problem.

Although alchemical mutation methods have been used in several case studies addressing drug resistance^49–51^, their prediction performance has not been compared to that of other FEP methods. In this study, we demonstrated that MutationFEP overcomes the intrinsic drawbacks of MP-CAFEE, a double-annihilation method. Therefore, choosing the proper FEP method based on the mutation type appears to be essential for rapid and accurate prediction of mutation-induced changes in drug sensitivity. For example, MutationFEP successfully reproduced the experimental ΔΔ*G* values resulting from several double point mutations in NA (i.e., H275Y/S247N and H275Y/I223V), for which ΔΔ*G* predictions appear to be challenging due to higher degrees of perturbation compared to single point mutations. Therefore, MutationFEP is suitable for examining the effects of both single and multiple mutations leading to drug resistance^5,52^. However, because MutationFEP cannot accommodate deletion/insertion mutations and replacement of proline or cysteine involved in a disulfide bond^23^, conventional FEP methods such as MP-CAFEE are more suitable for predictions of ΔΔ*G* associated with these types of mutations^16^. It is expected that these FEP methods will be further improved in the near future to actualize computer-assisted precision medicine.

## Conclusion

In this study, an MD-based alchemical mutation method, MutationFEP, and a traditional alchemical free energy computation method, MP-CAFEE, were compared in terms of performance at predicting mutation-induced changes in drug sensitivity. In three protein target systems, MutationFEP showed better prediction performance. Even though two of the systems included double point mutants, MutationFEP successfully reproduced experimental drug-sensitivity changes, suggesting that this protocol may be useful for assessment of the effects of multiple mutations, which are often found in drug-resistant cancer cells^5,52^. Also, its moderate perturbation scheme appears to be applicable to protein targets for which drug tends to leave the pocket during conventional FEP simulations. However, MutationFEP cannot currently handle mutation types other than amino acid replacements (i.e., deletions/insertions). Therefore, for realization of computer-assisted precision medicine, combined use of MutationFEP and traditional FEP methods is expected to cover a broader range of protein targets and mutation types. Further studies are needed to improve the computational methods to accurately predict mutation-induced drug sensitivity changes. Sample scripts, input coordinates, and parameters of MutationFEP are available as Supplemental File.

## Supplementary Information

Supplementary Information.

Supplementary Information.
