# Supplementary material for: Improvement in predicting drug sensitivity changes associated with protein mutations using a molecular dynamics based alchemical mutation method
Source: Sci Rep. 2020 Feb 7;10:2161. doi: 10.1038/s41598-020-58877-9 (PMC7005789; doi:10.1038/s41598-020-58877-9)
Supplement: Supplementary file 1 — Supplementary Information. [file 41598_2020_58877_MOESM1_ESM.docx]

Improvement in predicting drug sensitivity changes associated with protein mutations using a molecular dynamics based alchemical mutation method

Fumie Ono ^1^, Shuntaro Chiba ^2^ , Yuta Isaka ^3^, Shigeyuki Matsumoto ^2^, Biao Ma ^3^, Ryohei Katayama ^4^, Mitsugu Araki ^1^, Yasushi Okuno ^1,2,3*^

**^1^** Graduate School of Medicine, Kyoto University, 53 Shogoin-Kawaharacho, Sakyo-ku, Kyoto, Japan

^2^ Medical Sciences Innovation Hub Program, RIKEN, 1-7-22, Suehiro-cho, Tsurumi-ku, Kanagawa, Japan

^3^ Research and Development Group for In Silico Drug Discovery, Center for Cluster Development and Coordination (CCD), Foundation for Biomedical Research and Innovation at Kobe (FBRI) 6-3-5, Minatojima-Minamimachi Chuo-ku, Kobe Japan, Hyogo, Japan

^4^ Division of Experimental Chemotherapy, Cancer Chemotherapy Center, Japanese Foundation for Cancer Research, Tokyo, Japan

***** Corresponding author

Email: okuno.yasushi.4c@kyoto-u.ac.jp (YO)

# Supplemental Information


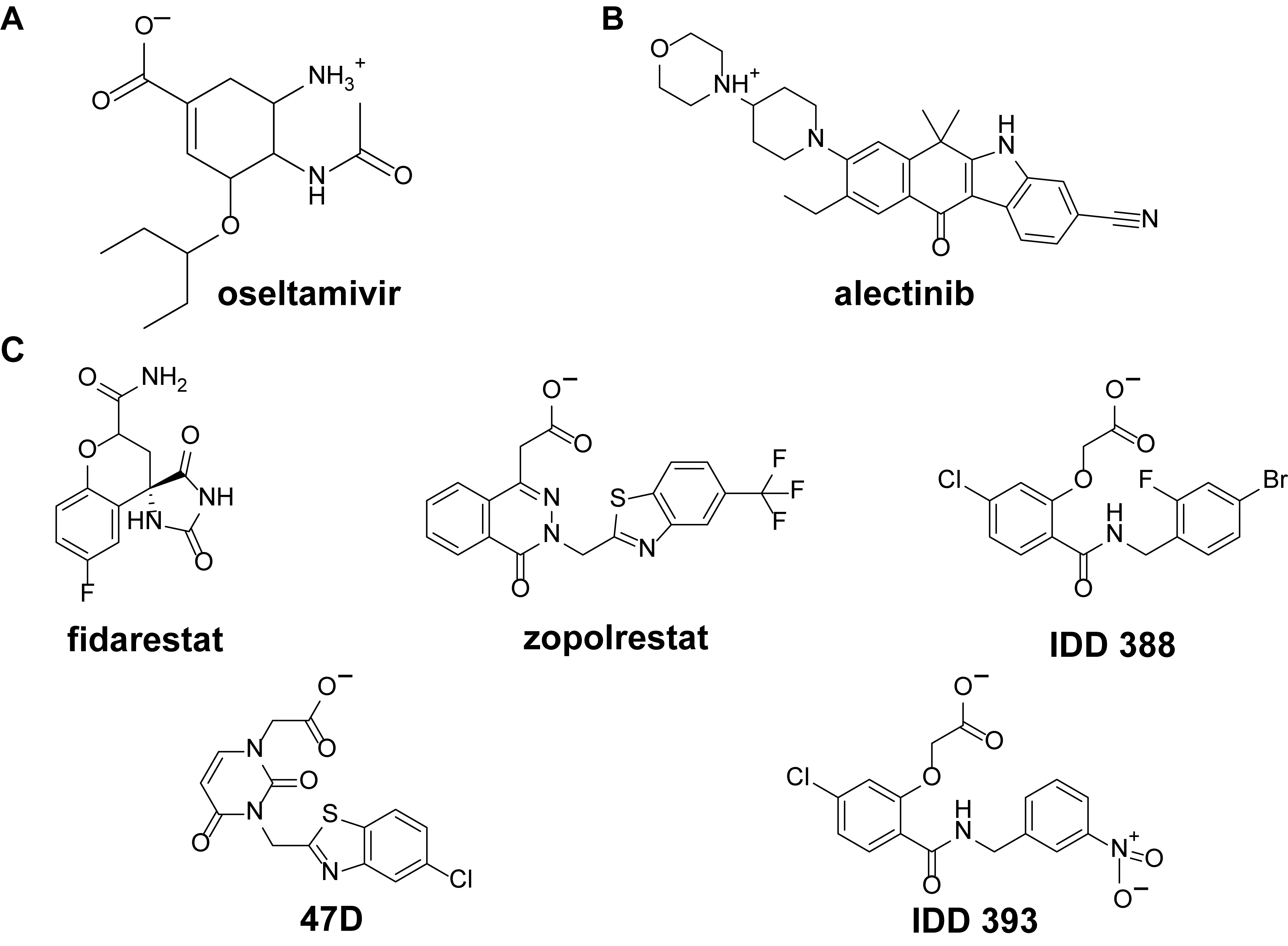


**Fig. S1.** The drugs used in simulation for (A) NA-oseltamivir, (B) ALK-alectinib, and (C) ALR2-drug systems.

**Table S1. *λ* values used for MutationFEP calculations.**

| Total number of *λ* | 27 *λ* | 14 *λ* | 8 *λ* |
| --- | --- | --- | --- |
| Value of each *λ* | 0 | 0 | 0 |
|  | 0.0125 |  |  |
|  | 0.025 | 0.025 |  |
|  | 0.0375 |  |  |
|  | 0.05 | 0.05 | 0.05 |
|  | 0.1 |  |  |
|  | 0.15 | 0.15 |  |
|  | 0.2 |  |  |
|  | 0.25 | 0.25 | 0.25 |
|  | 0.3 |  |  |
|  | 0.35 | 0.35 |  |
|  | 0.4 |  |  |
|  | 0.45 | 0.45 | 0.45 |
|  | 0.5 |  |  |
|  | 0.55 | 0.55 |  |
|  | 0.6 |  |  |
|  | 0.65 | 0.65 | 0.65 |
|  | 0.7 |  |  |
|  | 0.75 | 0.75 |  |
|  | 0.8 |  |  |
|  | 0.85 | 0.85 | 0.85 |
|  | 0.9 |  |  |
|  | 0.95 | 0.95 |  |
|  | 0.9625 |  |  |
|  | 0.975 | 0.975 | 0.975 |
|  | 0.9875 |  |  |
|  | 1 | 1 | 1 |

**
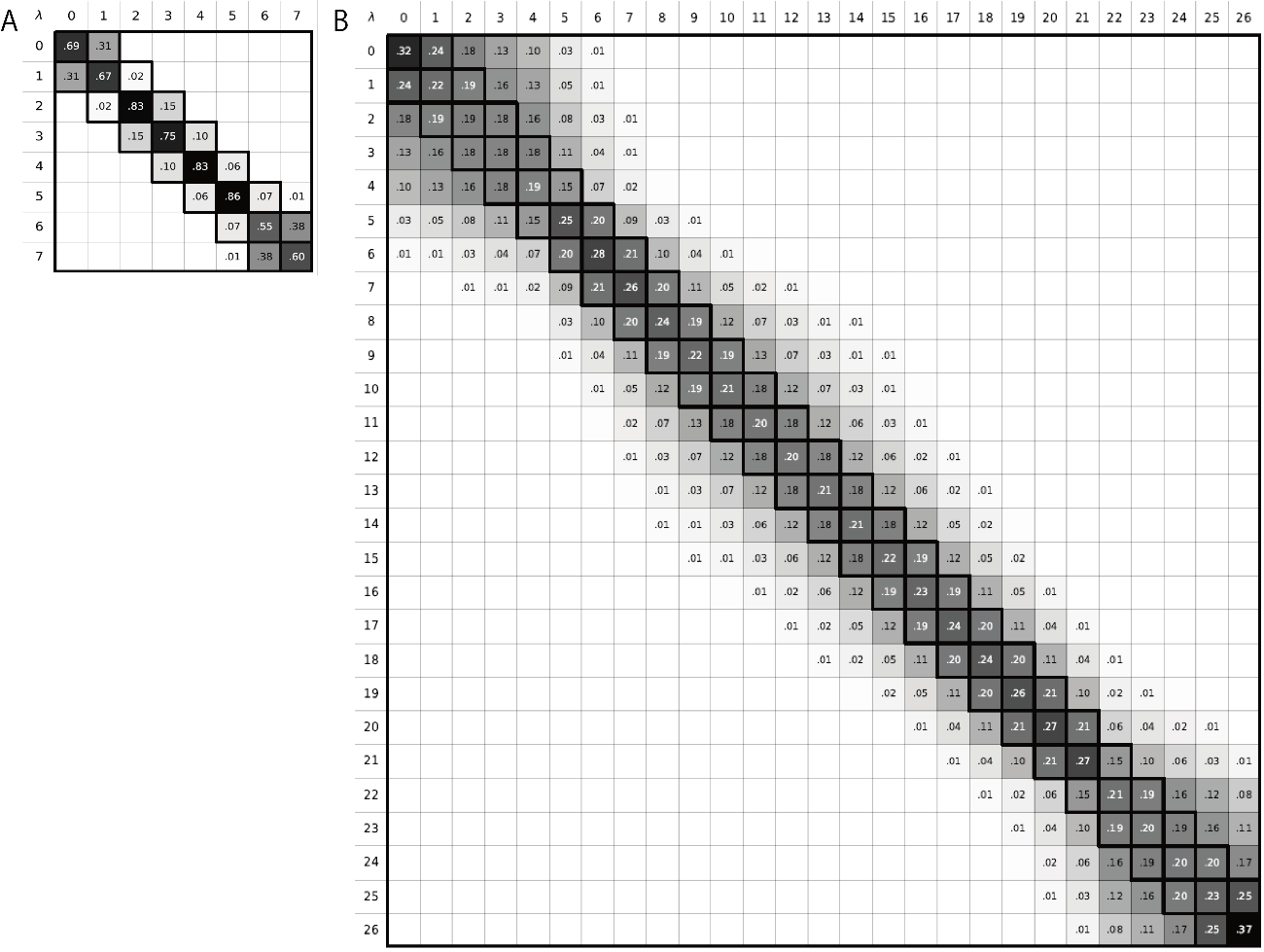
**

**Figure S2.** Energy overlap between *λ*s. (A) H275Y: the number of *λ* = 8 and the length of simulation = 5 ns. The energy overlap between the first and second *λ*s is 0.02, which can cause marked increase in the calculation error. (B) H275Y: the number of *λ* = 27 and the length of simulation = 5 ns.

**Table S2A.** Experimental and calculated drug-sensitivity change of NA-oseltamivir.

| Mutation | Experimental value*^a^* ($\text{Δ}\text{Δ}\text{G}$)  (kcal/mol) | Calculated value (${\text{Δ}\text{Δ}\text{G}}_{\text{MutationFEP}}$)*^b^* (kcal/mol) | | | | Calculated value*^c^*  (${\text{Δ}\text{Δ}\text{G}}_{\text{MP-CAFFE}}$) (kcal/mol) |
| --- | --- | --- | --- | --- | --- | --- |
|  |  | 2 − 3 ns  27 *λ^d^* | 2 − 5 ns  27 *λ* | 2 − 5 ns  14 *λ* | 2 − 5 ns  8 *λ* |  |
| I223V | 2.02 | 0.095 ± 0.03 | 0.071 ± 0.09 | 0.021 ± 0.16 | 0.034 ± 0.20 | -0.54 ± 1.14 |
| S247N | 1.76 | -0.49 ± 0.20 | -0.71 ± 0.25 | -0.91 ± 0.86 | 0.57 ± 1.37 | 2.34 ± 2.28 |
| H275Y/S247N | 5.47 | 3.28 ± 0.68 | 2.60 ± 0.33 | 2.33 ± 0.73 | 3.18 ± 1.74 | 4.61 ± 1.35 |
| H275Y/I223V | 4.96 | 1.59 ± 0.60 | 1.23 ± 0.79 | 0.34 ± 1.10 | 0.77 ± 0.85 | 0.34 ± 1.61 |
| H275Y | 4.22 | 1.80 ± 0.70 | 1.61 ± 0.79 | 1.81 ± 1.36 | 2.56 ± 1.63 | 1.16 ± 1.98 |

*a*: Experimental inhibition constants retrieved from the literature ^1^.

*b*: Drug-affinity change is calculated via ${{\text{Δ}\text{Δ}\text{G}}_{\mathrm{MutationFEP}}=\text{Δ}\text{G}}_{\text{mutation}}^{\text{2}}\text{ }\text{−}\text{ }\text{∆}\text{G}_{\text{mutation}}^{\text{1}}$. The uncertainty is defined as $\sqrt{\left( \text{σ}_{\text{mutation}}^{\text{1}} \right)^{\text{2}}\text{ }\text{+}\text{ }\left( \text{σ}_{\text{mutation}}^{\text{2}} \right)^{\text{2}}}$, where $\text{σ}_{\text{mutation}}^{\text{1}}$ and$\text{σ}_{\text{mutation}}^{\text{2}}$ represent the standard deviation of Δ$\text{G}_{\text{mutation}}^{\text{1}}$ and $\text{Δ}\text{G}_{\text{mutation}}^{\text{2}}$, respectively, across three independent simulations.

*c*: Drug-affinity change is calculated via ${\text{Δ}\text{G}}_{\text{MP-CAFEE}}\text{ }\text{=}\text{ }{\text{Δ}\text{G}}_{\text{bind}}^{\text{2}}\text{ }\text{−}{\text{ }\text{Δ}\text{G}}_{\text{bind}}^{\text{1}}$. The uncertainty is defined as the standard deviation of ${\text{Δ}\text{G}}_{\text{bind}}^{\text{2}}$ across the six independent simulations.

*d*: The sampling simulation time and the number of *λ* for the free energy calculations are given.

**Table S2B.** Experimental and calculated drug-sensitivity change of ALK-alectinib.

| Mutation | Experimental value*^a^*  ${\text{(}\text{RT}\text{ }\text{log(IC}}_{\text{50}}^{\mathrm{mut}}\text{ }\text{/}\text{ }\text{IC}_{\text{50}}^{\text{wt}})$) | Calculated value (${\text{Δ}\text{Δ}\text{G}}_{\text{MutationFEP}}$)*^b^* (kcal/mol) | | | | Calculated value*^c^*  (${\text{Δ}\text{Δ}\text{G}}_{MP-CAFFE}$) (kcal/mol) |
| --- | --- | --- | --- | --- | --- | --- |
|  |  | 2 − 3 ns  27 *λ^d^* | 2 − 5 ns  27 *λ* | 2 − 5 ns  14 *λ* | 2 − 5 ns  8 *λ* |  |
| F1174V | 0.66 | -0.25 ± 0.44 | 0.00 ± 0.37 | 0.09 ± 0.50 | 0.13 ± 0.21 | 2.20 ± 1.63 |
| I1171N | 2.07 | -0.24 ± 0.88 | 0.35 ± 0.18 | 0.71 ± 0.43 | 0.47 ± 0.93 | 3.08 ± 0.55 |
| L1196M | 1.28 | -0.29 ± 0.14 | -0.41 ± 0.06 | -0.52 ± 0.18 | -0.61 ± 0.34 | 1.57 ± 0.85 |
| F1174I | 0.12 | 0.44 ± 0.40 | 0.01 ± 0.22 | 0.28 ± 0.52 | 0.69 ± 0.85 | 2.16 ± 1.18 |
| V1185L | 0.16 | -0.59 ± 0.66 | -0.50 ± 0.44 | -0.07 ± 0.91 | -0.27 ± 0.48 | 1.88 ± 0.85 |
| G1269A | 0.51 | 0.09 ± 0.16 | 0.18 ± 0.22 | 0.36 ± 0.31 | 0.04 ± 0.19 | 1.02 ± 0.84 |
| V1180L | 1.46 | 0.41 ± 0.21 | 0.65 ± 0.23 | 0.93 ± 0.31 | 0.65 ± 0.67 | 0.45 ± 0.61 |
| L1196Q | 2.06 | 2.07 ± 0.32 | 2.40 ± 0.22 | 2.27 ± 0.17 | 2.47 ± 0.33 | 1.84 ± 0.60 |
| I1171T | 0.73 | -0.27 ± 0.33 | -0.22 ± 0.29 | -0.04 ± 0.72 | 1.03 ± 0.40 | 0.86 ± 0.83 |

*a*: IC_50_ values of alectinib for ALK mutants in the cell viability assays are retrieved from the literature ^2^. Those for ALK wild-type, G1269A, and F1174I mutants were determined by the identical procedure in this study.

*b*: Drug-senstivity change is calculated via $\text{Δ}\text{Δ}\text{G}\text{MutationFEP}{\text{ }\text{=}\text{ }\text{Δ}\text{G}}_{\text{mutation}}^{\text{2}}\text{ }\text{−}\text{ ∆}\text{G}_{\text{mutation}}^{\text{1}}$. The uncertainty is defined as $\sqrt{\left( \text{σ}_{\text{mutation}}^{\text{1}} \right)^{\text{2}}\text{ }\text{+}\text{ }\left( \text{σ}_{\text{mutation}}^{\text{2}} \right)^{\text{2}}}$, where $\text{σ}_{\text{mutation}}^{\text{1}}\text{ and}\text{ }\text{σ}_{\text{mutation}}^{\text{2}}$ represent the standard deviation of Δ$\text{G}_{\text{mutation}}^{\text{1}}$ and $\text{Δ}\text{G}_{\text{mutation}}^{\text{2}}$, respectively, across three independent simulations.

*c*: Drug-sensitivity change is calculated via ${\text{Δ}\text{G}}_{\text{MP-CAFEE}}\text{ }\text{=}\text{ }{\text{Δ}\text{G}}_{\text{bind}}^{\text{2}}\text{ }\text{−}\text{ }{\text{Δ}\text{G}}_{\text{bind}}^{\text{1}}$. The uncertainty is defined as the standard deviation of ${\text{Δ}\text{G}}_{\text{bind}}^{\text{2}}$ across the six independent simulations.

*d*: The sampling simulation time and the number of *λ* for the free energy calculations are given.

**Table S2C.** Experimental and calculated drug-sensitivity change of ALR2 with 5 drugs.

|  | Mutation | Experimental value*^a^*  ($\text{Δ}\text{Δ}\text{G}$) | Calculated value*^c^* (ΔΔ*G*_MutationFEP_) (kcal/mol) | | Calculated value*^c^*  (${\text{Δ}\text{Δ}\text{G}}_{MP-CAFFE}$) (kcal/mol) |
| --- | --- | --- | --- | --- | --- |
|  |  |  | 2 − 3 ns  27 *λ^d^* | 2 − 5 ns  27 *λ^d^* |  |
| ZST | V47I | 0.86 | -0.48 ± 0.10 | -0.45 ± 0.11 | 2.47 ± 1.11 |
|  | T113Y | 3.05 | 11.90 ± 1.16 | 11.20 ± 0.98 | 1.57 ± 0.70 |
|  | L300A | 1.07 | -0.21 ± 0.21 | -0.24 ± 0.23 | 1.16 ± 0.76 |
|  | L301M | 1.19 | -0.06 ± 0.25 | -0.15 ± 0.12 | -2.10 ± 0.84 |
|  | S302R/C303D | 3.31 | 12.31 ± 3.08 | 11.64 ± 2.43 | 5.31 ± 1.14 |
| FID | V47I | 0.14 | 0.29 ± 0.20 | 0.36 ± 0.09 | 3.01 ± 0.94 |
|  | T113Y | 2.05 | -0.14 ± 1.24 | -0.28 ± 1.00 | -0.41 ± 1.03 |
|  | L300A | 0.45 | -0.16 ± 0.54 | 0.07 ± 0.37 | -2.56 ± 0.55 |
|  | L301M | 0.69 | -0.04 ± 0.11 | 0.00 ± 0.12 | -1.15 ± 0.56 |
|  | S302R/C303D | 1.88 | -0.12 ± 4.54 | 0.42 ± 4.12 | 1.09 ± 0.79 |
| 388 | V47I | -0.31 | 7.52 ± 1.22 | 0.26 ± 0.12 | -1.43 ± 0.69 |
|  | T113Y | 2.05 | 0.23 ± 0.13 | 7.17 ± 0.96 | 7.39 ± 1.89 |
| 47D | V47I | 1.19 | 0.64 ± 0.10 | 0.58 ± 0.11 | -2.00 ± 1.49 |
|  | T113Y | 2.57 | 6.28 ± 0.98 | 6.81 ± 0.69 | 12.79 ± 1.60 |
|  | L300A | -0.24 | -1.17 ± 0.27 | -1.26 ± 0.17 | 0.40 ± 0.79 |
|  | S302R/C303D | 2.71 | 11.13 ± 3.82 | 10.31 ± 3.88 | 6.44 ± 0.97 |
| 393 | T113Y | 2.31 | 7.84 ± 0.36 | 8.07 ± 0.45 | 5.19 ± 0.92 |
|  | L300A | 0.45 | 0.45 ± 0.43 | 0.79 ± 0.48 | -8.82 ± 0.59 |
|  | S302R/C303D | 2.45 | 7.89 ± 1.22 | 7.56 ± 1.99 | -14.14 ± 1.54 |

*a*: ΔΔ*G* values for ALR2 mutants with 5 drugs are retrieved from the literature^3^.

*b*: Drug-sensitivity change is calculated via $\text{Δ}\text{Δ}\text{G}\text{MutationFEP}{\text{ }\text{=}\text{ }\text{Δ}\text{G}}_{\text{mutation}}^{\text{2}}\text{ }\text{−}\text{ ∆}\text{G}_{\text{mutation}}^{\text{1}}$. The uncertainty is defined as $\sqrt{\left( \text{σ}_{\text{mutation}}^{\text{1}} \right)^{\text{2}}\text{ }\text{+}\text{ }\left( \text{σ}_{\text{mutation}}^{\text{2}} \right)^{\text{2}}}$, where $\text{σ}_{\text{mutation}}^{\text{1}}\text{ and}\text{ }\text{σ}_{\text{mutation}}^{\text{2}}$ represent the standard deviation of Δ$\text{G}_{\text{mutation}}^{\text{1}}$ and $\text{Δ}\text{G}_{\text{mutation}}^{\text{2}}$, respectively, across three independent simulations.

*c*: Drug-sensitivity change is calculated via ${\text{Δ}\text{G}}_{\text{MP-CAFEE}}\text{ }\text{=}\text{ }{\text{Δ}\text{G}}_{\text{bind}}^{\text{2}}\text{ }\text{−}\text{ }{\text{Δ}\text{G}}_{\text{bind}}^{\text{1}}$. The uncertainty is defined as the standard deviation of ${\text{Δ}\text{G}}_{\text{bind}}^{\text{2}}$ across the six independent simulations.

*d*: The sampling simulation time and the number of *λ* for the free energy calculations are given.

# References

1. Pokorná, J. *et al.* Kinetic, thermodynamic, and structural analysis of drug resistance mutations in neuraminidase from the 2009 pandemic influenza virus. *Viruses* **10**, 339 (2018).

2. Okada, K. *et al.* Prediction of ALK mutations mediating ALK-TKIs resistance and drug re-purposing to overcome the resistance. *EBioMedicine* **41**, 105–119 (2019).

3. Steuber, H., Heine, A., Podjarny, A. & Klebe, G. Merging the Binding Sites of Aldose and Aldehyde Reductase for Detection of Inhibitor Selectivity-determining Features. *J. Mol. Biol.* **379**, 991–1016 (2008).
